# Serum Catestatin Level as a Stratification Assessment Tool in Non-Critical COVID-19 Patients

**DOI:** 10.3390/ijerph20021136

**Published:** 2023-01-09

**Authors:** Ivan Jerkovic, Vedran Kovacic, Tina Ticinovic Kurir, Josko Bozic, Leida Tandara

**Affiliations:** 1 Department for Urgent and Intensive Medicine with Clinical Pharmacology and Toxicology, Internal Medicine Clinic, University Hospital Split, University of Split School of Medicine, 21000 Split, Croatia; 2 Department of Endocrinology, Internal Medicine Clinic, University Hospital Split, University of Split School of Medicine, 21000 Split, Croatia; 3 Department of Pathophysiology, University of Split School of Medicine, 21000 Split, Croatia; 4 Department of Medical Laboratory Diagnostics, University Hospital Split, University of Split School of Medicine, 21000 Split, Croatia

**Keywords:** catestatin, SARS-CoV-2 infection, COVID-19

## Abstract

Introduction: Catestatin (CST) is a peptide with immunomodulatory, anti-inflammatory, and anti-microbial activities. There are only a few studies that have investigated plasma CST levels in COVID-19 patients (mostly in ICU patients). In our work, the aim was to demonstrate serum CST levels and their correlation with clinical outcomes in a group of severe COVID-19 patients admitted to the non-ICU department. Methods: The subjects were 32 patients (25 females, 7 males) admitted to the non-ICU unit for COVID-19 patients. Results: CST levels in our cohort were higher (8.91 ± 7.00) than previously reported CST levels in control subjects. We found a significant positive correlation between serum CST levels and C-reactive protein (r = 0.423, *p* = 0.008), D-dimers (r = 0.395, *p* = 0.013), hsTNT (high-sensitivity troponin T) (r = 0.603, *p* < 0.001), proBNP (N-terminal pro-brain natriuretic peptide) (r = 0.569, *p* < 0.001), and hospitalization days (r = 0.388, *p* = 0.014). There was a difference between groups of participants with SOFA <3 (*n* = 18) and SOFA >=3 (*n* = 14) in catestatin serum levels (7.25 ± 3.66 vs. 11.05 ± 9.52 ng/mL), but the difference was statistically insignificant (*p* = 0.065). Conclusion: We considered plasma CST level at hospital admission as a possible tool for early risk assessment in non-critical COVID-19 patients. This study is an attempt to clarify the complex pathophysiological mechanisms present in the development of severe forms of SARS-CoV2 infection.

## 1. Introduction

**Figure 1:**
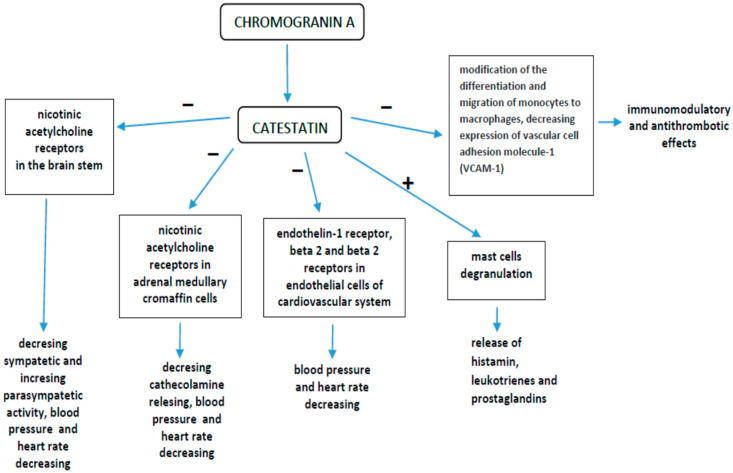
Catestatin mechanisms of action with clinical effects.

Catestatin (CST) is a human peptide produced from the chromogranin A cleavage process in the endocrine, nervous, and immune systems [1]. CST is an endogenous inhibitor of the nicotinic cholinergic receptor and inhibits catecholamine secretion while stimulating histamine release [2]. The biological effects of CST include the activation of polymorphonuclear white blood cells [3], the modulation of inflammation [4], and antimicrobial activities in multiresistant bacterial infections [5] and fungal infections [6]. Additionally, CST could be involved in inflammatory regulation in many clinical settings or be the target of anti-inflammatory treatment (Figure 1) [7].

Many publications have demonstrated a positive correlation between catestatin serum level and chronic inflammatory processes such as inflammatory bowel disease [8], rheumatoid arthritis, and systemic lupus erythematosus [9]. Several studies have demonstrated the influence of CST on vasodilatation [10], insulin resistance, and endothelial dysfunction, as well as its effect on the cardiovascular system in general [11].

Coronavirus disease 2019 (COVID-19) is an infectious disease caused by SARS-CoV-2 (severe acute respiratory syndrome coronavirus 2) and is associated with several clinical aspects and pathological manifestations ranging from mild flu-like disease to fatal multiple organ failure and respiratory failure [12]. SARS-CoV-2 primarily affects the lungs, but it can also induce acute myocardial injury, myocarditis, renal failure, and thromboembolic incidents [13]. For example, pulmonary involvement can progress from acute lung injury to fatal acute respiratory distress syndrome, and the risk factors and possible pathophysiological mechanisms of such an escalation of systemic inflammation are still unrecognized or insufficiently known [14]. Some possible markers of COVID-19 progression could be increased levels of C-reactive protein or the high release of pro-inflammatory cytokines [15]. 

CST, as an important immunomodulatory component, could also be involved in the regulation of severe systemic inflammatory conditions resembling those that occur as a result of COVID-19 infection [16]. As overt inflammatory response and the suppression of COVID-19-specific immune responses occur during severe COVID-19 infection, a need for the investigation of CST in severe COVID-19 infections arises. Besides inflammation, the additional pathophysiological connection between CST and severe COVID-19 infection is endothelial dysfunction. As severe COVID-19 can very often present as life-threatening thromboembolism and rates of thrombosis are substantially increased in COVID-19 hospitalized patients, it is possible that CST affecting the vascular system through suppression of endothelial dysfunction might be involved in the pathophysiology of COVID-19-associated thrombosis among hospitalized COVID-19 patients [17]. Consequently, serum CST levels could be a prognostic marker of the severity of COVID-19.

Despite numerous studies and significant scientific interest in COVID-19, there is a lack of information regarding the correlation between plasma CST levels and the clinical course of COVID-19.

There are only a few studies that have investigated plasma CST levels in COVID-19 patients (mostly in ICU patients), and these studies revealed that COVID-19 patients release significant amounts of CST in the plasma and that CST might predict poor COVID-19 outcomes. De Lorenzo et al. [18] reported that plasma chromogranin A (precursor of CST) levels were increased in COVID-19 patients and represented an early independent predictor of mortality. In the study, a significant number of participants were admitted to ICUs (21%). Schneider et al. [19] demonstrated that plasma CST could help in predicting in-hospital mortality among COVID-19 patients, with most of the participants being ICU patients. A similar conclusion that serum catestatin could be a predictor of poor outcomes was reported in a cohort of ICU COVID-19 patients [20].

To date, there have been no studies that have correlated clinical outcomes and serum CST levels at admission among non-ICU hospitalized COVID-19 patients. This report aimed to estimate serum CST levels and demonstrate their correlation with clinical parameters in a group of severe but non-critical COVID-19 patients admitted to a non-ICU department. The present study was conducted on the hypothesis that CST is involved in the pathophysiology of COVID-19 and that admission measurement of CST could help clinicians in terms of risk assessments of COVID-19 patients.

## 2. Materials and Methods

The Ethical Committee of the University Hospital of Split approved this study. The study was conducted according to the principles of the Declaration of Helsinki.

### 2.1. Subjects

The subjects were patients admitted during the second surge of COVID-19 in April and May 2020 to a non-ICU unit for COVID-19 patients (high-dependency unit) in the Infectology Department of University Hospital Split, Croatia. The inclusion criteria were as follows: age ≥18 years, radiologic findings of COVID-19 pneumonia, and COVID-19 positivity confirmed by a nucleic acid test. Exclusion criteria were disseminated malignancy or severe co-infection. Finally, the study included 32 subjects (25 females, 7 males (21.9%)). Arterial hypertension was previously diagnosed in 27 participants, chronic heart failure in 12, diabetes mellitus in 9, chronic kidney disease in 2, and chronic obstructive pulmonary disease in 4. None of the subjects needed mechanical ventilation, and in case of significant hypoxia, oxygen was administered via nasal prongs or masks. All patients survived and were discharged from the hospital without the need for ICU admission during hospitalization.

### 2.2. Laboratory Analysis

Blood samples were collected according to standard laboratory practice on hospital admission day, and standard laboratory parameters were analyzed. Samples of catestatin were stored at −80 °C and analyzed over the next few days. COVID-19 diagnosis was based on a positive real-time reverse transcriptase-polymerase chain reaction (RT-PCR) from a nasopharyngeal swab. An enzyme-linked immunosorbent assay (EK-053–27CE, EIA kit, Phoenix Pharmaceuticals Inc., Burlingame, CA, USA) was used for assessment of serum CST levels. According to the manufacturer’s declaration, the measurement range for CST was 0–100 ng/mL. The reported sensitivity of the assay kit to catestatin was 0.05 ng/mL, with a linear range of 0.05–0.92 ng/mL and 100% cross-reactivity with endogenous human catestatin (intra-assay and inter-assay coefficients of variability were <10% and <15%, respectively). All blood samples were analyzed in the same certified institutional biochemical laboratory using standard operating procedures. 

### 2.3. Statistical Analysis

The results were expressed as the arithmetic mean ± the standard deviation. Correlations between variables were tested by Pearson’s correlation coefficients. A linear regression model with a plot was constructed. The significance of differences in the mean between the two groups was assessed by unpaired Student’s *t*-tests. A multiple regression model (enter method) was constructed for analysis of the correlations of multiple variables. Statistical analysis was performed with SPSS software for Windows (IBM SPSS Statistics for Windows, version 26.0, Armonk, NY, USA). A one-tailed *p*-value < 0.05 was considered statistically significant.

## 3. Results

Demographic, clinical, and laboratory characteristics of the patients (*n* = 32) are summarized in Table 1. The mean age of participants was 80.50 ± 9.86 years. Serum CST levels in the overall cohort were 8.91 ± 7.00 ng/mL.

**Figure 2:**
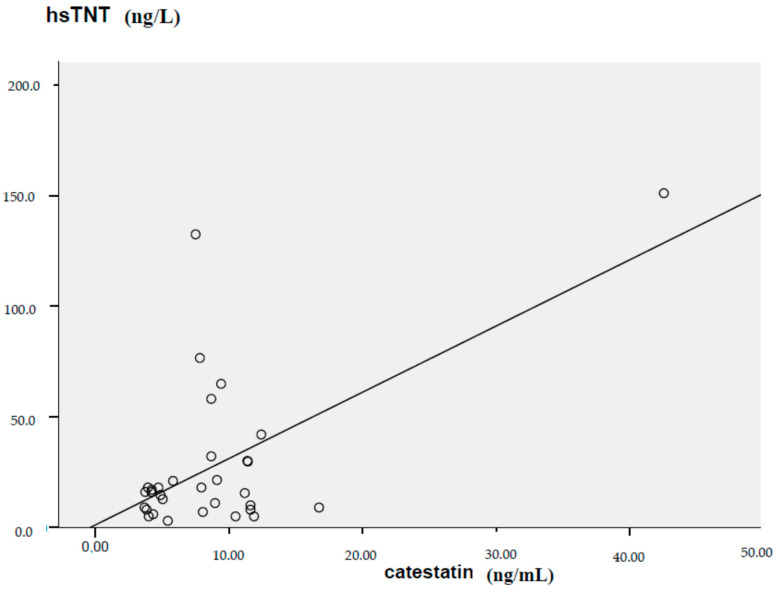
Linear regression plot between high-sensitivity troponin T and serum catestatin concentration (Y = 2.99X + 1.17).

We found a significant positive correlation between serum CST levels and C-reactive protein (r = 0.423; *p* = 0.008), D-dimers (r = 0.395; *p* = 0.013), hsTNT (high-sensitivity troponin T) (r = 0.603; *p* < 0.001), proBNP (N-terminal pro-brain natriuretic peptide) (r = 0.569; *p* < 0.001), and hospitalization days (r = 0.388, *p* = 0.014). The correlation between high-sensitivity troponin T and catestatin serum concentration is demonstrated in Figure 2 as a linear regression plot and is presented alongside its calculated regression equation.

The correlation between cardiovascular injury markers and clinical measures of the subjects is demonstrated in Table 2. Both hsTNT (high-sensitivity troponin T) and proBNP (N-terminal pro-brain natriuretic peptide) were positively correlated with COVID-19 severity (measured as SOFA score, MEWS score, or hospitalization days).

Differences between groups of subjects in CST and inflammatory and cardiovascular parameters are demonstrated in Table 3, Table 4 and Table 5. There was a difference between groups with SOFA <3 (n = 18) and SOFA >=3 (n = 14) in plasma CST levels (7.25 ± 3.66 vs. 11.05 ± 9.52 ng/mL), though the difference did not reach statistical significance (*p* = 0.065). hsTNT (high-sensitivity troponin T) and proBNP (N-terminal pro-brain natriuretic peptide) were significantly higher in groups of subjects with higher modified SOFA scores and less oxygen saturation, as well as among subjects treated with oxygen therapy.

A multiple regression model was constructed between SOFA score as a dependent variable and cardiovascular injury and inflammatory markers as predictors (Table 6). In this estimated regression model, catestatin, CRP, and proBNP were significant predictors of SOFA score.

## 4. Discussion

To our knowledge, this is the first study to report on serum CST levels among a cohort of non-critical COVID-19 patients and the results confirm that CST levels could have some role as a clinical prognostic parameter among non-ICU COVID-19 patients. We demonstrated a correlation between CST and severity scores and outcomes of non-critical COVID-19. Additionally, we found a strong correlation between CST and markers of cardiovascular injury, such as high-sensitivity troponin T (hsTNT) and N-terminal pro-brain natriuretic peptide (proBNP). Overall, CST levels in our cohort were higher than previously reported CST levels in control subjects [19] but significantly lower than COVID-19 subjects admitted to ICUs in a previous report [20]. 

In hospitalized non-critical COVID-19 patients, systemic inflammation is not as dominant as it is in critical COVID-19 patients who are treated in the ICU, and some of them have very severe systemic inflammation with cytokine storms [21]. In non-critical COVID-19 subjects, inflammation could be subtle, and novel markers of cardiovascular injury (or inflammation) might help clinicians in the stratification of such patients. A possible pathophysiological link between CST and the outcome of COVID-19 is an increased level of endothelial dysfunction, and endothelial function could be correlated with CST levels. As thromboembolism is the hallmark of COVID-19 [22], patients with increased plasmatic CST levels could be at risk of embolic incidents. Thus, plasmatic CST in non-critical COVID-19 patients could be included in risk estimation with possible clinical implications. Additionally, in our study, plasmatic levels of hsTNT and proBNP were correlated with COVID-19 severity and emphasized the importance of cardiovascular injury markers as risk estimation tools for phenotypes of COVID-19 with low-grade inflammation in non-ICU settings. The strong correlation between CST and hsTNT in our group of subjects confirms the hypothesis supporting the important cardiovascular effects of CST in relatively stable COVID-19 patients and confirms the role of CST in the complex pathophysiological pathways of COVID-19. 

Previous publications have reported COVID-19-related outcomes based on inflammation, endothelial dysfunction, and coagulopathy, as these events play a major role in the pathophysiology of the SARS-CoV2 virus [23]. CST and other molecules included in neuroendocrine activation are less studied.

De Lorenzo and et al. [18] recently reported that chromogranin A (but not vasostatin I) was significantly increased in patients who died from COVID-19 compared to survivors. They concluded that chromogranin A represents an early independent predictor of mortality. Chromogranin A in previous studies was increased in non-COVID ICU patients and has important prognostic value [24]. Chromogranin A is cleaved by proteases such as prohormone convertases, furin, cathepsin, plasmin, and thrombin to generate biologically active fragments [25], such as vasostatin I or CST.

CST is an endogenous multifunctional neuroendocrine peptide and might be more largely involved in the neural regulation of immunity [26]. By its biological function, CST is a specific, non-competitive, nicotinic cholinergic antagonist with inhibitory activity on catecholamine secretion induced by nicotine that results from blockages to the flux of Na+ and Ca^2+^ from the extracellular space to the cytosol [27]. CST activates human mast cells through the degranulation and release of several leukotrienes and prostaglandins, suggesting that CST might have an immunomodulatory function [28]. The immunomodulatory properties of CST have been demonstrated in an animal model, where CST increased thrombus resolution by attenuating endothelial inflammation, suggesting it might provide a novel approach for anti-thrombosis treatment [29]. Accepting the immunomodulatory and antithrombotic properties of CST, it is not unexpected to find increased plasmatic CST levels in many inflammatory diseases [30]. Previous reports on the effects of catestatin on the endothelial, cardiovascular, and immunological systems are summarized in Table 7.

To date, there have been very few reports on plasmatic CST levels in COVID-19 patients, and the vast majority of subjects in those studies were ICU-admitted critical COVID-19 patients. Kljakovic-Gaspic et al. [20] in an ICU-based study found that serum CST levels were high in both fatal and non-fatal COVID-19 ICU-treated patients, despite CST levels being significantly lower in the fatal group than the non-fatal group. They concluded that serum CST was a significant negative predictor of fatal COVID-19 outcomes, and CST could have a major role in the pathophysiology of SARS-CoV-2 infections.

Schneider et al. [19] reported significantly higher admission plasma CST concentrations in a group of COVID-19 positive patients with respiratory failure (SpO2 < 90%) than in a group of COVID-19 negative participants. In their study, compared to healthy controls, COVID-19 positive patients had severe hypoxemia with hyperinflammatory profiles. They concluded that COVID-19 patients with respiratory failure release significant amounts of CST in the plasma, thus making this protein available for the neural regulation of immunity. Additionally, CST concentrations were significantly associated with in-hospital mortality, and plasma CST might thus reliably help in predicting in-hospital mortality.

We found a strong correlation between CST and markers of cardiovascular injury, such as high-sensitivity troponin T (hsTNT) and N-terminal pro-brain natriuretic peptide (proBNP). This finding contrasts with previous reports on the correlation between the plasmatic level of CST and the inhibition of atherosclerosis [31], probably due to the diminishing effect of CST on endothelial inflammation [32]. In terms of cardiovascular effects, CST is confirmed to be antihypertensive [33] and anti-inflammatory [34]. It is possible that complex SARS-CoV2 pathophysiological infection pathways may release CST to stabilize and balance neuro-immunological response to systemic virus pathogenicity. 

The systemic effect of COVID-19 can be explained by endothelial dysfunction and systemic inflammation. Namely, angiotensin-converting enzyme 2 (ACE2) is used as a receptor for SARS-CoV-2. Endothelial cells become infected by the virus due to ACE2 being expressed in the human endothelium, and the virus reduces the integrity of endothelial tissue, thus leading to the exposure of prothrombotic molecules, platelet adhesion, the activation of coagulation cascades, and, consequently, vascular damage [35]. The possible role of CST in non-fatal COVID-19 patients might be related to the downregulation of such endothelial vascular damage [36]. As a previous report [20] demonstrated that CST levels were significantly lower in the fatal group compared to the non-fatal group, plasmatic CST might only serve as a prognostic tool in COVID-19 stratification for less critical COVID-19 patients. In fatal/critical COVID-19 patients, flare ups and unbalanced and unregulated inflammation occur alongside cytokine storms, and in such clinical COVID-19 phenotypes, neuroendocrine regulation of systemic inflammation is diminished and overruled.

An additional possible explanation for higher CST levels in non-ICU patients with more severe COVID-19 is hypoxemia, as the hypoxic environment in COVID-19 patients with pulmonary infiltrates should augment the release of CST. This assumption is based on a previous report which demonstrated in an experimental model that hypoxia raises the levels of chromogranin A, a precursor of CST [37].

In conclusion, this study provides the first evidence of elevated plasma catestatin (CST) levels in a group of non-ICU admitted pulmonary COVID-19 patients. As CST levels were significantly increased in more severe COVID-19 infections, we considered plasma CST level at hospital admission as a possible tool for early risk assessments of non-critical COVID-19 patients. Considering immunological and endothelial damage due to COVID-19, this study is an attempt to clarify extremely complex pathophysiological mechanisms that lead to the development of severe forms of SARS-CoV2 infection.

Larger and more multicentric studies are needed to investigate CST as a mediator of immunity in severe viral infection and to elucidate its role in the complex pathophysiological neuroendocrine–immunological axis. It is of the utmost importance to confirm CST as a diagnostic or risk stratification tool and find out the possible therapeutic implications of CST and similar peptides in the neuroendocrinal regulation of systemic inflammation and endothelial function.

This study has some limitations. Cross-sectional design and a relatively small number of participants limits stronger causative conclusions. Additionally, the relatively old population included in the study could also have a significant impact on the results.

The major findings of this study are as follows:

- Plasma catestatin (CST) levels were elevated in a group of non-ICU pulmonary COVID-19 patients (lower than in COVID-19 subjects admitted to the ICU in a previous study).
- CST levels were correlated with severity score and outcome for non-critical COVID-19 patients.
- A strong correlation between CST and markers of cardiovascular injury was confirmed.
- CST level at hospital admission might be a possible tool for early risk assessments and may also be a clinical prognostic parameter in non-critical COVID-19 patients.

## 5. Conclusions

We confirmed elevated plasma catestatin (CST) levels in pulmonary non-critical COVID-19 patients. As CST levels were correlated with severity score and outcomes, CST could serve as novel prognostic marker in COVID-19 patients admitted in non-intensive care facilities. 

## Figures and Tables

**Table 1:** Characteristics of the subjects (*n* = 32).

	Mean ± Std. Deviation	Minimum	Maximum
catestatin (ng/mL)	8.91 ± 7.00	3.68	42.55
age (years)	80.50 ± 9.86	58.00	97.00
SOFA score	3.16 ± 2.41	1.00	12.00
MEWS score	2.59 ± 2.08	1.00	11.00
SatO2 (%)	92.06 ± 6.89	64.00	98.00
leukocytes (×10^9^/L)	6.63 ± 4.02	2.40	20.70
D-dimers (mg/L)	4.36 ± 3.85	0.20	15.41
urea (mmol/L)	8.43 ± 5.56	1.70	26.90
creatinine (μmol/L)	105.12 ± 140.58	20.00	829.00
albumins (g/L)	30.09 ± 5.85	21.00	43.00
proteins (g/L)	58.72 ± 7.99	46.00	78.00
procalcitonin (ng/mL)	0.15 ± 0.19	0.01	0.66
CRP (mg/L)	32.60 ± 40.76	0.60	195.70
LDH (IU/L)	211.13 ± 76.17	88.00	424.00
sodium (mmol/L)	136.41 ± 3.60	128.00	142.00
potassium (mmol/L)	3.94 ± 0.41	3.10	4.90
chloride (mmol/L)	100.38 ± 4.63	89.00	109.00
hsTNT (ng/L)	27.83 ± 34.78	3.00	151.00
proBNP (pg/mL)	3036.13 ± 5031.61	20.00	18882.00
heart rate (/min)	90.13 ± 24.58	55.00	180.00
systolic pressure (mmHg)	123.59 ± 27.45	73.00	195.00
diastolic pressure (mmHg)	77.56 ± 17.03	35.00	115.00
hospitalization (days)	30.59 ± 10.97	15.00	61.00

**Legend**: SatO2: oxygen saturation of hemoglobin in arterial blood; SOFA: Sequential Organ Failure Assessment; MEWS: Modified Early Warning Score; CRP: C-reactive protein; LDH: lactate dehydrogenase; hsTNT: high-sensitivity troponin T; proBNP: N-terminal pro-brain natriuretic peptide.

**Table 2:** The correlation between cardiovascular injury markers and clinical measures of the subjects (Pearson’s test, one-tailed).

	hsTNT		proBNP	
	r	*p*	r	*p*
hsTNT	/	/	**0.686**	**<0.001**
proBNP	**0.686**	**<0.001**	/	/
SOFA score	**0.336**	**0.030**	**0.556**	**<0.001**
MEWS score	**0.356**	**0.023**	**0.532**	**0.001**
leukocytes	0.247	0.087	**0.465**	**0.004**
neutrophils	**0.412**	**0.010**	**0.425**	**0.008**
lymphocytes	**−0.423**	**0.008**	**−0.0425**	**0.008**
D-dimers	**0.435**	**0.006**	**0.623**	**<0.001**
prothrombin time	−0.241	0.092	−0.189	0.149
urea	0.236	0.097	**0.442**	**0.006**
creatinine	0.189	0.150	**0.384**	**0.015**
albumins	−0.280	0.060	−0.240	0.093
procalcitonin	0.225	0.108	**0.432**	**0.007**
LDH	**0.349**	**0.025**	**0.312**	**0.041**
sodium	0.073	0.345	<0.001	0.500
potassium	0.109	0.276	0.086	0.320
systolic pressure	**−0.361**	**0.021**	**−0.475**	**0.003**
diastolic pressure	**−0.395**	**0.013**	**−0.457**	**0.004**
heart rate	0.277	0.062	**0.386**	**0.014**
hospitalization days	**0.408**	**0.010**	**0.453**	**0.005**

**Legend**: r: Pearson’s correlation coefficient; *p*: significance; SOFA: Sequential Organ Failure Assessment; MEWS: Modified Early Warning Score; LDH: lactate dehydrogenase; hsTNT: high-sensitivity troponin T; proBNP: N-terminal pro-brain natriuretic peptide; significant correlations (*p* < 0.05) are shown in bold.

**Table 3:** Differences between groups of participants with SOFA < 3 (*n* = 18) and SOFA >= 3 (*n* = 14) in CST and cardiovascular and inflammatory parameters (Student’s *t*-test, one-tailed).

	SOFA Score >= 3	SOFA Score < 3	
	Mean ± Std. Deviation	Mean ± Std. Deviation	*p*
catestatin (ng/mL)	11.05 ± 9.52	7.25 ± 3.66	0.065
procalcitonin (ng/mL)	0.21 ± 0.20	0.10 ± 0.17	0.052
hsTNT (ng/L)	43.76 ± 46.46	15.45 ± 13.54	0.010 *
proBNP (pg/mL)	5470.21 ± 6695.19	1142.94 ± 1792.74	0.007 *
albumins (g/L)	26.14 ± 3.55	33.17 ± 5.47	<0.001 *
CRP (mg/L)	48.92 ± 51.39	19.91 ± 24.90	0.022 *

**Legend**: SOFA: Sequential Organ Failure Assessment; CRP: C-reactive protein; hsTNT: high-sensitivity troponin T; proBNP: N-terminal pro-brain natriuretic peptide; *p*: significance; *: *p* < 0.05.

**Table 4:** Differences between participants with hemoglobin oxygen saturation > 94% (*n* = 18) and <94% (n = 14) in CST and cardiovascular and inflammatory parameters (Student’s *t*-test, one-tailed).

	SatO2 % >= 94	SatO2 % < 94	
	Mean ± Std. Deviation	Mean ± Std. Deviation	*p*
catestatin (ng/mL)	7.54 ± 4.03	10.67 ± 9.47	0.11
procalcitonin (ng/mL)	0.10 ± 0.15	0.21 ± 0.21	0.05
hsTNT (ng/L)	12.76 ± 9.12	47.22 ± 45.34	<0.001 *
proBNP (pg/mL)	1152.22 ± 2690.12	5458.29 ± 6298.88	0.01 *
albumins (g/L)	32.33 ± 5.77	27.21 ± 4.71	0.01 *
CRP (mg/L)	19.39 ± 24.92	49.59 ± 50.96	0.02 *

**Legend**: SatO2: oxygen saturation of hemoglobin in arterial blood; CRP: C-reactive protein; hsTNT: high-sensitivity troponin T; proBNP: N-terminal pro-brain natriuretic peptide; *p*: significance; *: *p* < 0.05.

**Table 5:** Differences between participants on oxygen therapy (*n* = 12) and participants without oxygen therapy during hospitalization (*n* = 20) in CST and cardiovascular and inflammatory parameters (Student’s *t*-test, one-tailed).

	No Oxygen Therapy	Oxygen Therapy	
	Mean ± Std. Deviation	Mean ± Std. Deviation	*p*
catestatin (ng/mL)	7.75 ± 3.91	10.85 ± 10.26	0.12
procalcitonin (ng/mL)	0.13 ± 0.18	0.17 ± 0.20	0.32
hsTNT (ng/L)	16.79 ± 16.92	46.24 ± 48.19	0.01 *
proBNP (pg/mL)	2115.05 ± 4418.59	4571.25 ± 5788.93	0.09
albumins (g/L)	31.70 ± 6.02	27.42 ± 4.62	0.02 *
CRP (mg/L)	28.43 ± 45.90	39.57 ± 30.95	0.23

**Legend**: CRP: C-reactive protein; hsTNT: high-sensitivity troponin T; proBNP: N-terminal pro-brain natriuretic peptide; *p*: significance; *: *p* < 0.05.

**Table 6:** Multiple regression model (enter method) between SOFA score as a dependent variable and CST, cardiovascular injury markers, and inflammatory markers as predictors.

	Beta	*p*
catestatin	−0.372	0.033 *
albumins	0.026	0.881
procalcitonin	0.242	0.166
CRP	−2.775	0.009 *
hsTNT	0.089	0.627
proBNP	0.641	0.002 *

**Legend**: CRP: C-reactive protein; hsTNT: high-sensitivity troponin T; proBNP: N-terminal pro-brain natriuretic peptide; *p*: significance; Beta: standardized coefficient; *: *p* < 0.05.

**Table 7:** Previous reports on the effects of catestatin on the endothelial, cardiovascular, and immunological systems.

Effects of Catestatin	Report Design	Pathophysiological and Clinical Implications	Reference
immunological	in vitro	activation of polymorphonuclear white blood cells	[3]
immunological	animal model	modulation of inflammation (attenuated expression of proinflammatory genes, increased expression of anti-inflammatory genes)	[4]
antimicrobial	in vitro	antibacterial activity	[5]
antifungal	in vitro	antifungal activity, possible treatment against Candida	[6]
immunological	review	shift in macrophage differentiation from a pro- to an anti-inflammatory phenotype	[7]
anti-inflammatory	cross-sectional observational	catestatin levels are higher in inflammatory bowel disease	[8]
cardiovascular	human experimental	catestatin dilates human blood vessels in vivo, especially in females	[10]
cardiovascular	review	effect on insulin resistance, endothelial dysfunction, hypertension	[11]
immunological	review	neuromodulation in inflammatory and autoimmune diseases	[27]
immunological	in vitro	catestatin activates human mast cells, peptide might have immunomodulatory functions	[28]
antithrombotic	animal model	catestatin increases thrombus resolution, attenuates endothelial inflammation	[29]
immunological	cross-sectional observational	catestatin is increased in rheumatoid arthritis patients	[30]
anti-inflammatory, cardiovascular	in vitro	catestatin prevents macrophage-driven atherosclerosis	[31]
anti-inflammatory, cardiovascular	review	reduce the risk of developing hypertension, antimicrobial effects	[32]
anti-inflammatory, cardiovascular	in vitro	protect the heart against excessive sympatho-chromaffin overactivation,	[33]
anti-inflammatory, cardiovascular	animal model	anti-inflammatory cardiac effects, remodeling of the myocardium	[34]

## Data Availability

The data presented in this study are available on request from the corresponding author.
